# Establishing Surgical Care Sustainability in Sub-Saharan Africa for Global Child Health: Insights From Pediatric Cardiac Surgical Capacity-Building Programs in Ethiopia and Côte d'Ivoire

**DOI:** 10.3389/fped.2021.806019

**Published:** 2022-01-14

**Authors:** Jayoung Park, Jongho Heo, Woong-Han Kim

**Affiliations:** ^1^Program in Global Surgery and Implementation Science, JW LEE Center for Global Medicine, Seoul National University College of Medicine, Seoul, South Korea; ^2^Department of Human Systems Medicine, Seoul National University College of Medicine, Seoul, South Korea; ^3^National Assembly Futures Institute, Seoul, South Korea; ^4^Department of Thoracic and Cardiovascular Surgery, Seoul National University Children's Hospital, Seoul National University College of Medicine, Seoul, South Korea

**Keywords:** global surgery, congenital heart disease, team-based, capacity-building, Sub-Saharan Africa

## Abstract

The global surgery research team of the JW LEE Center for Global Medicine, Seoul National University College of Medicine, introduced team-based health workforce training programs for pediatric cardiac surgery in Ethiopia and Côte d'Ivoire. A team-based collaborative capacity-building model was implemented in both countries, and details of the program design and delivery were documented. The research team shared their experiences and identified achievements, lessons, and challenges for cardiac surgical interventions in Sub-Saharan Africa. Future directions were put forward to advance and strengthen the low-and middle-income countries “Safe Surgery.”

## Introduction

At each step of the surgical journey of a patient in a hospital, holistic care cannot be provided unless health professionals have in-depth clinical knowledge and excel in non-technical skills such as teamwork (1). Teamwork involves collaboration to achieve common goals, and its characteristics include open communication, collaboration willingness, mutual performance monitoring, backup behavior, and team orientation (2). With effective teamwork, medical staff can share the common goals of providing quality care and ensuring a positive health outcome. This is also true for surgery. A single surgeon cannot perform surgery. Perioperative holistic care from various medical divisions needs to be provided for a patient to receive optimal surgery and live a healthy life afterward. Holistic care includes an accurate diagnosis; surgery by a well-trained and skilled surgeon; assistance for the surgeon by a surgical technician, operating room nurses, and ancillary services; and intensive care after surgery. The importance of teamwork in a hospital setting is well-described; however, the current understanding of hospital teamwork is exclusive to high-income countries (HICs), and the culture of surgical teamwork has not yet been widely established in low- and middle-income countries (3). This article aims to share our experiences with applying the team-based collaborative capacity-building model to workforce training programs for cardiac surgery and interventional cardiology in Ethiopia and Côte d'Ivoire for sustainable surgical care for children and identify achievements and limitations that will guide future cardiac surgical interventions in Sub-Saharan Africa.

## Introduction of a Team-Based Collaborative Capacity-Building Model

### Designing a Team-Based Model

A surgical team led by Dr. Woong-Han Kim of South Korea established a team-based collaborative capacity-building model for developing countries. The Korean team used the “sandwich program” as part of the team-based capacity-building model. The Sandwich program has been used by the Swedish International Development Cooperation Agency and other global fellowship programs, and it is well-known for connecting the step-by-step advancement of a fellow with the development of institutional capacity to build an enabling environment for professional growth, which enhances patient care (4). As part of the program, a group of Korean cardiac surgeons, pediatric cardiologists, anesthesiologists, perfusionists, intensive care unit nurses, administrative assistants, and researchers visit a host country. During the on-site visit, the Korean team organizes didactic and clinical seminars, discussions, and hands-on training. The Korean and local teams handle all aspects of diagnosis, surgical treatment, and pediatric intensive care. The team meets during morning and postoperative conferences to review the surgical procedure for children who have undergone open-heart surgery or interventional catheterization and talk about how to improve patient health. Didactics and clinical lectures are provided according to the needs of the host team. In contrast with other HIC mission teams, this capacity-building program was implemented using guiding principles. The first task is to identify patients who are untreated or have more complex cases so that the host team can perform complex surgical procedures collaboratively with the Korean team or independently, but under the supervision of the Korean team, depending on the complexity and familiarity of the cases. The second obligation is to encourage and promote local team building. Importantly, Korean team members transfer their knowledge and skills to the host teams through team-based activities. For invitational training, 9–10 trainees from the required divisions for cardiac service are invited to a HIC for a minimum of 2 months. The goal of invitational training is to allow trainees to gain in-depth academic knowledge in related fields while experiencing diverse and specialized cardiac and vascular surgery, interventional cardiology, and patient care. During training, trainees have the opportunity to attend and present at domestic and international academic conferences.

### Perceived Benefits

#### National Level

The establishment of a surgical system at the teaching hospital contributes to continuing care for congenital heart disease and helps to reduce the national disease burden. Furthermore, low-and middle-income countries (LMICs) teaching hospitals serve as representative cardiac centers across neighboring countries, where there is no cardiovascular center, and they are equipped with personnel with the most up-to-date clinical experience and up-to-date advanced technology, enabling surrounding nations to access specific health services.

#### Institution Level

The team-based model benefits local institutions in terms of human development and infrastructure expansion. The model includes training of trainers, which cascades knowledge and skills of the Korean faculty through local trainers and, eventually, within a health network across a local hospital (5). The model will increase the overall human resource level at a local hospital. Furthermore, since subspecialty care necessitates substantial infrastructure, other healthcare priorities benefit from its expansion. Improved intensive care unit capacity benefits all critical care patients and improves anesthesia for all surgical patients. Enhanced screening facilitates optimal community health care as well as primary care (6).

#### Individual Level

Individuals benefit from the team-based program; these include medical staff, as well as patients and caregivers. According to the 10-year team-based collaborative capacity-building program evaluation from 2010 to 2019, the average Risk Adjustment for Congenital Heart Surgery 1 score increased from 1.9 to 2.78, while the average preoperative weight of patients decreased by 2.8 kg from 13 kg in 2009 to 10.2 kg in 2019 (7). In-depth interviews with Uzbek and Korean medical staff, patients, and caregivers showed an improvement in the surgical capacity of Uzbek medical personnel, an improvement in attitudes toward patients and colleagues, and changes in the quality of life of patients and caregivers (7). Based on these findings in Uzbekistan, the team-based program proved to be effective at strengthening the pediatric cardiovascular disease treatment capacity of low- and middle-income countries (8).

## Implementing a Team-Based Collaborative Capacity-Building Model in Ethiopia

Since 2015, six cardiac surgery training sessions and two interventional cardiology training sessions in Ethiopia, and two visiting training courses in Korea have been conducted. This program has received financial support from the Korea Foundation for International Healthcare (KOFIH), a public-affiliated organization of the Ministry of Health and Welfare. In 2016, the Ethiopian counterpart organization changed from Myungsun Christian Hospital, a private hospital, to Tikur Anbessa Specialized Hospital (TASH), a public university teaching hospital under Addis Ababa University for long-term sustainability and impact.

### Rotation 1: First On-Site Visit

For approximately a week, ultrasound-based diagnoses were made, surgery was performed, and postoperative care was provided at the host institution (TASH). The ultrasound-based diagnoses were made during the first 2 to 3 days of training, and the final surgery target was chosen. Following that, 1–2 surgeries were performed per day for ~10 surgeries. During the same period, transcatheter treatment for several congenital heart diseases, such as atrial septal defect, patent ductus arteriosus, and pulmonary stenosis, was completed by a Korean pediatric cardiologist and a pediatrician. Local medical staff participation was maximized to the greatest extent possible by including local medical staff in all surgeries and postoperative intensive care processes, resulting in the utilization of local staff-led clinical skills rather than Korean team-led clinical skills. By doing so, local medical personnel were encouraged to participate in hands-on training. During the on-site training period, 2–3 lectures were provided for ~1 hour. The selection of topics was based on the needs of local personnel or at the discretion of the Korean medical staff. Morning conferences were conducted daily based on the ultrasound-based diagnosis and surgical records from the beginning to the end of the on-site training period. Through a conference based on the patient's surgical records and progress, Korean and Ethiopian medical staff discussed the treatment strategies and the expected progress.

### Rotation 2: Invitational Training

A total of nine trainees were chosen for invitational training after passing the competency assessment and interview conducted by Korean medical staff in its first on-site training: two thoracic surgeons, one anesthesiologist, one pediatrician, three intensive care nurses, one operating room nurse, and one perfusionist. The invited trainees attended the Seoul National University Hospital Pediatric Heart Center's weekly meeting and engaged in an actual patient care plan for 2 months to understand team dynamics in the HIC setting. All trainees participated in all lectures, regardless of workforce type, and this increased mutual understanding between the professions, which is vital for developing teamwork.

### Rotation 3: Second On-Site Visit

During the second on-site visit, the HIC team evaluated the ability of trainees to apply their newly acquired skills in their home country hospital setting. Under the guidance of the HIC team, the trainees led the procedure, and patient care during the training period. The local team was trained to ensure that they could operate without assistance from the HIC team.

## Implementing a Team-Based Collaborative Capacity-Building Model in Côte D'Ivoire

In response to a request and invitation from the Institute of Cardiology of Abidjan (ICA), which had previously worked with a Korean doctor assigned to Côte d'Ivoire as part of the Korea International Cooperation Agency (KOICA) medical staff dispatch program in January 2019, the surgical team from Korea visited the ICA and performed nine cardiac surgeries for children with heart disease and four patients with cardiac interventions together with the local medical staff, and ~50 patients underwent ultrasound examination. During the on-site visit, local medical staff involvement and engagement in surgery increased, and improvements in their attitudes were observed. While local medical staff actively engaged in the surgery and conducted surgery alongside Korean medical staff, extracorporeal membrane oxygenation was used for open-heart surgery for the first time since the hospital's founding. This provided a constructive incentive for local medical personnel by showing that treatment and care could be performed adequately with the available equipment. Clinical education and didactic workshops were organized, and expertise was revitalized through the distribution of related educational materials and the completion of assigned tasks with the Korean team. The local team and visiting team gathered every morning to improve interdisciplinary communication and patient outcomes. After the program implementation, opinions on the project's feasibility were gathered and coordinated through meetings with the hospital director and local medical staff. The program's direction was additionally discussed in an interview with potential stakeholders such as Côte d'Ivoire's Minister of Health for government funding and cooperation. The HIC and LMIC experts both addressed sustainable teamwork capacity-building programs in Côte d'Ivoire during a meeting with the representative of the KOICA Côte d'Ivoire office. Due to the pandemic of coronavirus disease in 2019 (COVID-19), both the invitational training and the second on-site training scheduled for the following year have been canceled. As an alternative, Korea team explored the possibility of launching an e-learning program with the local team.

## Lessons and Challenges

### Desirable Attributes for HIC and LMIC Teams

#### Required Competency for a HIC Team

##### Appropriate Expertise Levels

Members of the HIC team must have an appropriate level of expertise to translate knowledge to local staff. The widely held belief that “any help is better to none” or that “some surgery, no matter how professional, is preferable to none,” is misleading (9). Increased untreatable mortality will impose an unnecessary burden on an already overburdened and resource-constrained health care system (9, 10). Therefore, participation of competent qualified practitioners who can figure out how to use the materials at hand to provide the best care for their patients and teach evidence-based medicine is critical. Medical staff in HICs, particularly those in positions of leadership, should critically examine their knowledge base and practice in the field of global surgery.

##### Global Health Competency Attainment

Through strengthening global health competency, an HIC team must be prepared for the unexpected situation of a resource-limited surgical system. An HIC team may be unfamiliar with surgical diseases or presentations in hosting communities and their added complexity; visitors may lack understanding or preparation to deal with cultural and linguistic challenges in unfamiliar settings (11–13). Moreover, the HIC team is required to work in facilities that lack basic infrastructure such as water and electricity, as well as insufficient supplies, equipment, and ancillary services, making it difficult to practice within one's scope of practice. The level of discomfort can be overwhelming if HIC members arrive without prior global health experience or understanding.

##### Listen to the Frontline Surgical Providers in LMICs

The implementor should fulfill teaching, research, and service missions while giving the local frontline surgical provider a voice for their potential needs. Frontline health providers play a key role as brokers (usually as patient advocates) between patients and the health system at the point of care. Thus, understanding their perspectives and actions can strengthen the planning and provision of future surgical services (14).

##### Identify a Key Champion

Engaging local champions is one of the four critical components to make global health programs effective and sustainable, according to Howell et al. (15). The local champion, who believes strongly in the cause, will align himself or herself with the intervention and understand local ecosystems. Thus, the role of local champions in keeping everything on course should not be underestimated (16). Therefore, the program implementation needs to reach out to local champions who dedicate their efforts.

#### Required Competency for an LMIC Team

##### Team Leadership

The performance of team members depends on the team leader's behavior, knowledge, and interpersonal and leadership skills (17). A local champion (a surgeon) should be constantly conscious of the implications of keeping the entire healthcare team in a well-grounded context (18). Through the leadership of a “safe surgery champion,” the initiative will quickly gain momentum during the early stage of development.

#### Building Effective Partnerships

It should be emphasized that cooperation and successful development of programs can be achieved with the full support and interest of local governments, especially for capacity building and human resource development programs that require a long-term solution. A long-term program needs new sustainable funding and supportive administrative processes. Therefore, local government commitment should be allocated to a global surgery partnership to support these long-term placements.

Institutional university and hospital partnerships are also critical: a network of governments and organizations that have made an institutional commitment to foster effective and long-term academic partnerships. Collaboration between universities and teaching hospitals can promote research and advocacy to support progressive policy agendas, advance medical education, and retain healthcare workers in economically marginalized areas (19).

#### Essential Infrastructure and Equipment

During on-site visits, the HIC team frequently brings appropriate technology that may be unavailable or unaffordable at the host institution or country. These are intended to be used in future surgery cases; however, donated equipment frequently become inoperable. According to a WHO report on medical device donations, “only 10–30% of donated equipment becomes operational in developing countries” (20). Ultimately, the hospital must devise a reliable supply chain for critical surgical devices. For cardiac surgery, a stable cardiopulmonary bypass machine and an oxygenator for open-heart surgery must be continuously supplied (21). Further research into the development of supply chains and advanced health technology management systems in LMICs to maximize the impact of the surgical capacity-building program should be conducted.

## Future Directions

### Prepare for an Unprecedented Crisis: E-Learning Program

Because of the unprecedented pandemic, international travel was restricted, so direct face-to-face exchange between the Korean surgical team and the overseas surgical team was not possible. As a result, with the remarkable rise in e-learning, team-based training was undertaken remotely and in a virtual setting. Korean supervisors provided all online materials to satisfy the needs of local staff. In December 2020, an e-learning program on cardiac surgery and interventional cardiology was first offered to all medical staff at the TASH in Ethiopia. The program consists of ~37 lectures and discussion sessions, and case study simulations with 3D printing led by Korea's most experienced medical staff. The program ran for 8 weeks, and participants were required to watch five to seven lectures per week and take part in the case studies. Certificates were distributed at the end of the program to participants who had completed all the lectures.

With this sudden move away from face-to-face programs, online learning technology played a key role with promising outcomes. Educational didactics have strengthened, and this will remain a component of the team-based curriculum during the post-COVID-19 period. Additionally, the translation and subtitles aided in bridging the language gap between the local and Korean teams. However, there are alternatives to online programs. While 3D-printed simulator activities were provided for trainee clinical practices, there are still limitations related to not receiving direct hands-on training within the local healthcare setting. As a result, it is incumbent on global surgery practitioners to thoroughly find ways to fill the gap, as the online program will continue to exist after the pandemic.

### Developing Sustainable Academic Partnership: Academic Global Surgery

Academic institutions should facilitate academic cooperation by rebuilding bilateral education programs. To improve surgery in LMICs, the surgical education system must be strengthened. However, according to a rapid review of academic global surgery programs, the existing academic global surgery programs are highly skewed toward HIC residents (22), and concerns have been raised that similar opportunities for LMIC medical staff are not frequently available (12). Furthermore, LMIC medical personnel must acquire learning autonomy and eventually replace visiting faculty. To close the gap, HIC academic institutions and LMIC academic institutions should cooperate in the future to provide opportunities for advanced certificates or academic degrees.

As a result of our learnings in applying the team-based collaborative capacity-building model for cardiac surgery and interventional cardiology training programs in Ethiopia and Côte d'Ivoire, we were able to identify achievements and limitations for future cardiac surgical interventions in the sub-Saharan region. A team-based collaborative capacity-building strategy was presented, as well as lessons and challenges learned through insights from pediatric cardiac capacity-building programs in Ethiopia and Côte d'Ivoire. This research also suggests future directions for a team-based paradigm for pediatric cardiac surgery competence in developing countries.

## Data Availability Statement

The original contributions presented in the study are included in the article/supplementary material, further inquiries can be directed to the corresponding author/s.

## Author Contributions

All authors made substantial contributions to conception, design, participated in drafting the article, revising it critically for important intellectual contents, and gave final approval of the version to be submitted.

## Conflict of Interest

The authors declare that the research was conducted in the absence of any commercial or financial relationships that could be construed as a potential conflict of interest.

## Publisher's Note

All claims expressed in this article are solely those of the authors and do not necessarily represent those of their affiliated organizations, or those of the publisher, the editors and the reviewers. Any product that may be evaluated in this article, or claim that may be made by its manufacturer, is not guaranteed or endorsed by the publisher.
